# COVID-19 and Kawasaki syndrome: should we really be surprised?

**DOI:** 10.1017/S1047951120001432

**Published:** 2020-05-15

**Authors:** Rohit S. Loomba, Enrique Villarreal, Saul Flores

**Affiliations:** 1 Advocate Children’s Hospital, Chicago Medical School/Rosalind Franklin University of Medicine and Science, Chicago, IL, USA; 2 Tecnologico de Monterrey, Escuela de Medicina y Ciencias de la Salud, Monterrey, Nuevo, Leon, Mexico; 3 Texas Children’s Hospital, Baylor College of Medicine, Houston, TX, USA

**Keywords:** COVID, coronavirus, pandemic, inflammation, Kawasaki, myocarditis

## Abstract

A hyperinflammatory response to COVID-19 is being described in children. While this presents, and responds to management, similar to that of Kawasaki Disease it is being coined a new entity. But is it really? We explore how this phenomenon may be Kawasaki Disease with a new trigger.

Recent reports have described a hyperinflammatory response to the novel, pandemic coronavirus, COVID-19. This entity has been described to resemble Kawasaki Disease with some of these children displaying coronary artery changes characteristic of Kawasaki Disease.^1^ Though, this entity has generated large media attention, should the medical community be really surprised?

While the aetiology of Kawasaki Disease has been considered elusive for some time, there is substantial data pointing to a likely viral aetiology.^2–5^ Many have hypothesised that some children may be genetically predisposed to a more robust inflammatory response to specific viruses. Once exposed to the specific virus, children then mount this exaggerated inflammatory response which clinically manifests as what is now defined to be Kawasaki Disease. Data from the Taiwanese national database have demonstrated the link of viral illness to Kawasaki Disease as have multiple other studies and case reports.^6^ One study even was able to isolate novel, viral RNA from cytoplasmic inclusion bodies in children with Kawasaki Disease, further demonstrating a viral association. Viruses associated with Kawasaki Disease include Influenza, Enterovirus, Adenovirus, Parvovirus, Rhovirus, respiratory synctitial virus, Varicella, Epstein-Barr, measles, and dengue.^7–21^ Association with previous coronavirus has also been demonstrated.^22^ With this in mind why should Kawasaki Disease with COVID-19 come as a surprise?

Additionally, hyperinflammatory response to COVID-19 has been reported at length in adults. Elevated inflammatory and reactive markers include C-reactive protein, procalcitonin, ferritin, and D-dimer among others. In fact, these have prognostic value as more ill COVID-19 patients have higher values. In adults, accumulating evidence suggests that a subgroup of patients with severe COVID-19 have a “*cytokine storm syndrome*” in which a cascade of activated cytokines leads to harmful auto-amplifying hyperinflammatory cytokine production.^23,24^ Adult patients with worse illness and greater evidence of inflammation also had a higher incidence of cardiac findings such as troponin leak and ventricular dysfunction. Thus, these findings also may simply be inherent to more severe COVID-19 secondary to overall inflammatory response. This has been noted in viremia from other agents as well, particularly in the form of myocarditis.

While characterising the effects of COVID-19 is important, we also must put these findings in context of what is previously known in relation to viruses and critical illness. We must be vigilant to not inappropriately create new clinical entities or exaggerate previous clinical entities simply because of association with COVID-19. For those meeting criteria of Kawasaki Disease or presenting like myocarditis, the diagnosis should be Kawasaki Disease and myocarditis, as appropriate, and not a new clinical diagnosis; and furthermore, clinical management should be as such. Future effort should continue to focus on determining the reasons for the coronary artery involvement and the virus tropism for the myocardium.

## References

[1] Ripenhagen SG , Gomez X , Gonzalez-Martinez C , Wilkinson N , Paraskevi T . Hyperinflammatory shock in children during COVID-19 pandemic. Lancet 2020. doi: 10.1016/S0140-6736(20)31094-1. PMC7204765 32386565

[2] L’Huillier AG , Brito F , Wagner N , et al. Identification of viral signatures using high-throughput sequencing on blood of patients with Kawasaki disease. Front Pediatr 2019; 7: 524. 31921732 10.3389/fped.2019.00524 PMC6930886

[3] Pitzer VE , Burgner D , Viboud C , et al. Modelling seasonal variations in the age and incidence of Kawasaki disease to explore possible infectious aetiologies. Proc Biol Sci 2012; 279: 2736–2743. 22398170 10.1098/rspb.2011.2464 PMC3367771

[4] Rowley AH , Baker SC , Shulman ST , et al. RNA-containing cytoplasmic inclusion bodies in ciliated bronchial epithelium months to years after acute Kawasaki disease. PLOS One 2008; 3: e1582. 18270572 10.1371/journal.pone.0001582 PMC2216059

[5] Giray T , Bicer S , Kucuk O , et al. Four cases with Kawasaki disease and viral infection: aetiology or association. Infez Med 2016; 24: 340–344. 28011972

[6] Chang LY , Lu CY , Shao PL , et al. Viral infections associated with Kawasaki disease. J Formos Med Assoc 2014; 113:148–154. 24495555 10.1016/j.jfma.2013.12.008 PMC7125523

[7] Hu P , Guan Y , Fan XC , Lu FY , Song LM. Incomplete Kawasaki disease induced by measles in a 6-month-old male infant. Int J Dermatol 2016; 55:e34–e36. 26518488 10.1111/ijd.13122

[8] Huang X , Huang P , Zhang L , et al. Influenza infection and Kawasaki disease. Rev Soc Brasde Med Trop 2015; 48: 243–248. 10.1590/0037-8682-0091-2015 26108000

[9] Guleria S , Jindal AK , Pandiarajan V , Singh MP , Singh S. Dengue-triggered Kawasaki disease: a report of 2 cases. J Clin Rheumatol 2018; 24: 401–404. 29424760 10.1097/RHU.0000000000000704

[10] Joob B , Wiwanitkit V. Kawasaki disease in a 2-year-old child with dengue fever: correspondence. Ind J Pediatr 2016; 83: 1054. 10.1007/s12098-015-2017-7 26806205

[11] Ferraz C , Cunha F , Mota TC , Carvalho JM , Simoes JS , Aparicio JM. Acute respiratory distress syndrome in a child with human parvovirus B19 infection. Pediatr Infect Dis J 2005; 24: 1009–1010. 16282940 10.1097/01.inf.0000183768.84890.ae PMC2896321

[12] Shike H , Shimizu C , Kanegaye JT , et al. Adenovirus, adeno-associated virus and Kawasaki disease. Pediatr Infect Dis J 2005; 24: 1011–1014. 16282942 10.1097/01.inf.0000183769.31951.1e

[13] Wang J , Sun F , Deng HL , Liu RQ. Influenza A (H1N1) pdm09 virus infection in a patient with incomplete Kawasaki disease: a case report. Medicine 2019; 98: e15009. 30985646 10.1097/MD.0000000000015009 PMC6485757

[14] Thissen JB , Isshiki M , Jaing C , et al. A novel variant of torque teno virus 7 identified in patients with Kawasaki disease. PLOS One 2018; 13: e0209683. 30592753 10.1371/journal.pone.0209683 PMC6310298

[15] Turkay S , Odemis E , Karadag A. Kawasaki disease onset during concomitant infections with varicella zoster and Epstein-Barr virus. J Natl Med Assoc 2006; 98: 1350–1352. 16916136 PMC2569579

[16] Maggio MC , Cimaz R , Alaimo A , Comparato C , Di Lisi D , Corsello G. Kawasaki disease triggered by parvovirus infection: an atypical case report of two siblings. J Med Case Rep 2019; 13: 104. 31014402 10.1186/s13256-019-2028-5 PMC6480815

[17] Sopontammarak S , Promphan W , Roymanee S , Phetpisan S. Positive serology for dengue viral infection in pediatric patients with Kawasaki disease in southern Thailand. Circ J 2008; 72: 1492–1494. 18724028 10.1253/circj.cj-08-0158

[18] Jordan-Villegas A , Chang ML , Ramilo O , Mejias A. Concomitant respiratory viral infections in children with Kawasaki disease. Pediatr Infect Dis J 2010; 29: 770–772. 20354462 10.1097/INF.0b013e3181dba70b PMC2927322

[19] Weng KP , Cheng-Chung Wei J , Hung YM , et al. Enterovirus infection and subsequent risk of Kawasaki disease: a population-based cohort study. Pediatr Infect Dis J 2018; 37: 310–315. 28834956 10.1097/INF.0000000000001748

[20] Jagadeesh A , Krishnamurthy S , Mahadevan S. Kawasaki disease in a 2-year-old child with dengue fever. Ind J Pediatr 2016; 83: 602–603. 10.1007/s12098-015-1927-8 26546113

[21] Catalano-Pons C , Quartier P , Leruez-Ville M , et al. Primary cytomegalovirus infection, atypical Kawasaki disease, and coronary aneurysms in 2 infants. Clin Infect Dis 2005; 41: e53–e56. 16080076 10.1086/432578

[22] Esper F , Shapiro ED , Weibel C , Ferguson D , Landry ML , Kahn JS. Association between a novel human coronavirus and Kawasaki disease. J Infect Dis 2005; 191: 499–502. 15655771 10.1086/428291 PMC7199489

[23] Jacobs JP , Stammers AH , St Louis J , et al. Extracorporeal membrane oxygenation in the treatment of severe pulmonary and cardiac compromise in COVID-19: experience with 32 patients. ASAIO J 2020. doi: 10.1097/MAT.0000000000001185. PMC7217117 32317557

[24] Mehta P , McAuley DF , Brown M , et al. COVID-19: consider cytokine storm syndromes and immunosuppression. Lancet 2020; 395: 1033–1034. 32192578 10.1016/S0140-6736(20)30628-0 PMC7270045

